# *In vitro* dynamic perfusion of prevascularized OECs-DBMs (outgrowth endothelial progenitor cell - demineralized bone matrix) complex fused to recipient vessels in an internal inosculation manner

**DOI:** 10.1080/21655979.2022.2085560

**Published:** 2022-06-22

**Authors:** Zhian Chen, Dixin Cai, Rongmao Shi, Wei Ding, Yongqing Xu, Hongbo Tan

**Affiliations:** a Graduate School, Kunming Medical University, Kunming, Yunnan, China; b Department of Orthopaedics, People’s Liberation Army Joint Logistic Support Force 920th Hospital, Kunming, Yunnan, China

**Keywords:** Dynamic perfusion culture, outgrowth endothelial progenitor cells, decalcified bone matrix, vascularization, internal inosculation

## Abstract

The current research on seed cells and scaffold materials of bone tissue engineering has achieved milestones. Nevertheless, necrosis of seed cells in center of bone scaffold is a bottleneck in tissue engineering. Therefore, this study aimed to investigate the *in vivo* inosculation mechanism of recipient microvasculature and prevascularized outgrowth endothelial progenitor cells (OECs)-demineralized bone matrix (DBM) complex. A dorsal skinfold window-chamber model with tail vein injection of Texas red-dextran was established to confirm the optimal observation time of microvessels. OECs-DBM complex under static and dynamic perfusion culture was implanted into the model to analyze vascularization. OECs-DBM complex was harvested on 12th day for HE staining and fluorescent imaging. The model was successfully constructed, and the most appropriate time to observe microvessels was 15 min after injection. The ingrowth of recipient microvessels arcoss the border of OECs-DBM complex increased with time in both groups, and more microvessels across the border were observed in dynamic perfusion group on 3rd, 5th, 7th day. Fluorescent integrated density of border in dynamic perfusion group was higher at all-time points, and the difference was more significant in central area. Fluorescent imaging of OECs-DBM complex exhibited that no enhanced green fluorescent protein-positive cells were found beyond the verge of DBM scaffold in both groups. *In vitro* prevascularization by dynamic perfusion culture can increase and accelerate the blood perfusion of OECs-DBM complex obtained from recipient microvasculature by internal inosculation. Accordingly, this approach may markedly contribute to the future success of tissue engineering applications in clinical practice.

## GRAPHICAL ABSTRACT

**Figure:**
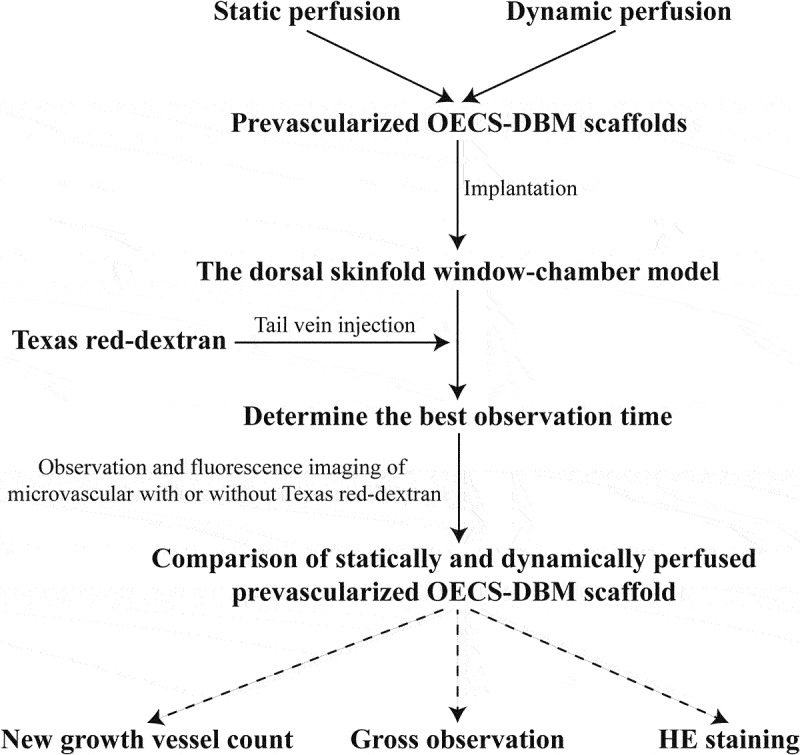

## Highlights

- Successful construction of the dorsal skinfold window-chamber model.
- The number of inosculation vessels increases faster in dynamic perfusion group.
- Prevascularized OECS-DBM fuses with recipient vessels via internal inosculation.

## Introduction

At present, large segmental bone defects are still a major clinical challenge in orthopedics. Bone tissue engineering is one of the ways to solve this dilemma. There are many strategies to promote vascularization of tissue-engineered bone, including the slow release of pro-vascular growth-related factors such as vascular endothelial growth factor (VEGF) [1–4], *in vivo* prevascularization [5–7] and *in vitro* prevascularization [8–10]. The critical factor for survival and function of tissue-engineered bone after implantation is rapid access to adequate blood perfusion [11]. With this in mind, many researchers have focused on the use of pro-vascular growth-related factors to stimulate angiogenesis in the recipient region and on the ingrowth of tissue-engineered products [2,4]. However, studies have confirmed that the limit of human microvascular growth rate is about 5 μm/h in physiological state [12,13]. It can be seen that it takes some time for the new blood vessels to ingrowth the scaffolds, and the cells in the central area of the scaffolds are exposed to ischemia and hypoxia during this time, resulting in necrosis of the seed cells in the pre-implantation period of tissue-engineered bone.

For this reason, the field of tissue engineering has gradually developed prevascularization techniques. The basic concept of prevascularization technology is to pre-build a microvascular network on a tissue-engineered product before it is implanted into the body. Its advantage is that the tissue-engineered product does not have to wait for the recipient vessels to grow in slowly after implantation, but only needs the microvascular network on the scaffolds to inosculation with the recipient vessels [14,15], so that sufficient blood perfusion and nutritional support from the recipient area can be obtained within a short period of time. *In vitro* prevascularization can be achieved by growing endothelial cells (ECs) on a scaffold. ECs have the potential to spontaneously form blood vessels [14,16]. The difficulties are the availability of autologous vascular ECs and the limited *in vitro* amplification capacity. Allogeneic-derived vascular ECs are more difficult to apply due to their immunogenicity, and the density of cells is not easily regulated when seeded into the scaffold. Peripheral blood-derived outgrowth endothelial cells (OECs), as cells with proliferative and differentiation potential toward mature ECs, are an important source of autologous vascular ECs, and widely available and easy to obtain. As precursors of mature ECs, OECs have cell morphology similar to mature ECs, express partially similar cell surface markers, and more importantly, have superior proliferative potential and angiogenic capacity [17,18]. OECs can not only tolerate low-temperature storage but also maintain the stability of cell phenotype and gene expression after high-fold amplification [19]. Peripheral blood OECs have become an important seed cell in tissue engineering because of their strong proliferative capacity and convenient source. Only a small number of cell clones are required to obtain sufficient numbers of cells for tissue engineering, and there are already many examples of their application to tissue engineering research [20–22].

Our previous studies have demonstrated that a dynamic perfusion system administering continuous fluid shear stress to OECs on demineralized bone matrix (DBM) enhances spreading adhesion, proliferation, differentiation to mature ECs, and three-dimensional tube formation in matrix pores, contributing to *in vitro* prevascularization of DBM [22]. Based on this, this study intends to observe the vascularization process and the possible fusion mechanism of OECs-DBM scaffolds with recipient vessels in real time after implantation into nude mice in static and dynamic perfusion culture by establishing a dorsal skinfold window-chamber model.

## Materials and methods

### Construction of the dorsal skinfold window-chamber model and implantation of OECs-DBM scaffolds

**Figure 1.**
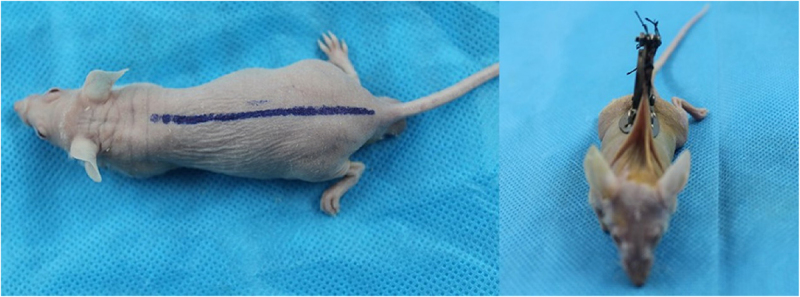
Dorsal skinfold window-chamber assembling on BABL/C nude mice.

**Figure 2.**
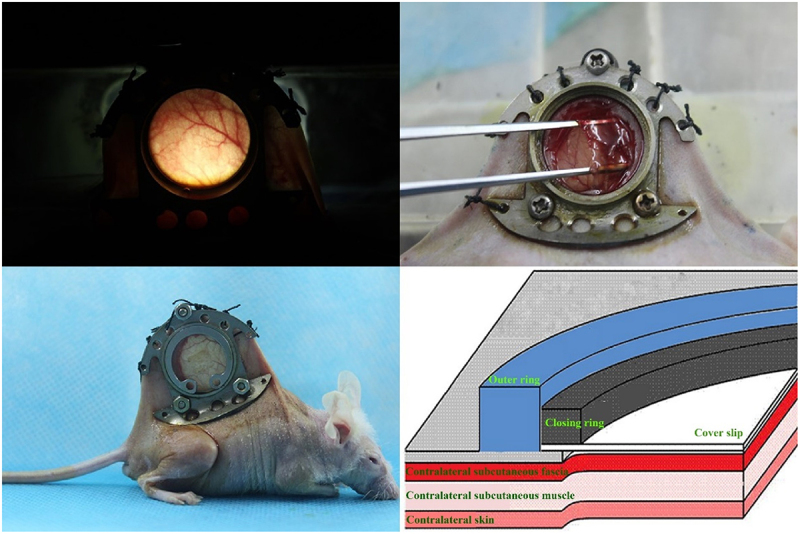
Implanting of OECs-DBM complex and illustration of implanting layers.

Isolation and identification of OECs, preparation of OECs-DBM scaffolds and static and dynamic perfusion cultures of OECs-DBM scaffolds were performed as previously described [22]. BALB/C male nude mice were purchased from Beijing Vital River Laboratory Animal Technology Co., Ltd., at 6–8 weeks, weighing range of 19–21 g. Nude mice were randomly divided into two groups of six each, and implanted with OECs-DBM scaffolds in the static culture group and dynamic perfusion group, respectively. The dorsal skinfold window-chamber model is constructed as described previously [23]. Briefly, pentobarbital (0.01 ml/g; Sigma-Aldrich, St. Louis, MO, USA) was administered intraperitoneally for the anesthesia of nude mice. Ophthalmic scissors are carefully cut through the skin of the mice’s dorsum until the vascularization dynamic viewing window (15 mm in diameter) is fully open. The window was rinsed with sterile saline and covered with a circular coverslip to close the window. After the nude mice recovered their diet and vitality and successfully passed the traumatic shock period, the closing ring and circular coverslip were removed. The OECs-DBM scaffold was implanted, and the fascia was covered, and then the circular coverslip and closing ring to close the window. Each OECs-DBM scaffold was implanted at the same site of nude mice, and the window was sterilized with iodophor from time to time. The animal experiments were performed as shown in Figure 1 and Figure 2. The above animal experiments were approved by the Animal Ethics Committee of Kunming Medical University.

### Microvascular fluorescence visualization

**Figure 3.**
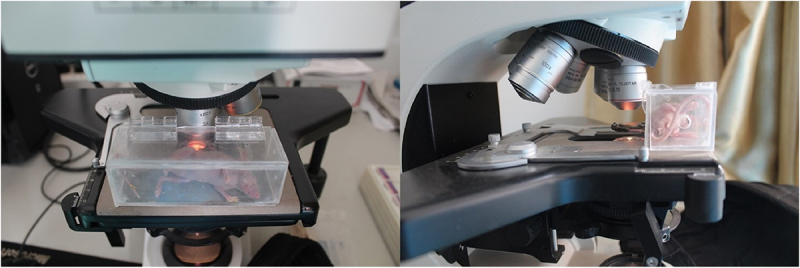
Observation platform for nude mice burdening dorsal skinfold window-chamber.

As shown in Figure 3, nude mice were fixed on a homemade observation stand and moved under a fluorescent microscope (Olympus, Tokyo, Japan). According to the instructions, Texas red-dextran (Invitrogen, Carlsbad, CA, USA) was formulated in sterile water to a concentration of 0.5 mg/ml, and Plasma fluorescent labeling was performed by tail vein injection of nude mice at a dose of 0.01 ml/g body weight. The microvessels were observed under the fluorescence microscope, and the starting time of microvessel development, the best observation time, and the duration of development were recorded. During the observation process, the microvessels were switched to the light microscope (Olympus, Tokyo, Japan) every 5 min and compared with the microvessel development under the fluorescence microscope. Image J software (version 1.0, National Institutes of Health) was utilized to measure the diameter of vessels that were not observed under light microscopy and were visualized under fluorescence microscopy.

### Dynamic observation of the vascularization process of OECs-DBM scaffolds

Nude mice were immobilized on homemade observation stands on days 1, 3, 5, 7, 9, and 12 after scaffold implantation. Texas red-dextran liquid was injected approximately 15 min prior to performing the observation. The observation stand was moved to the fluorescence microscope and imaged and taken pictures.

The number of ingrowth vessels at OECs-DBM scaffold border was detected. In a nutshell, the enhanced green fluorescent protein-labeled green fluorescent cells were first observed under a fluorescent microscope (excitation wavelength 488 nm, emission wavelength 597 nm) to determine the extent of the OECs-DBM scaffold edge, and then the vessels were searched and counted along the border of the OECs-DBM scaffolds (excitation wavelength 595 nm, emission wavelength 620 nm). The criteria for new growth into the vessel at the edge of the scaffolds were that it did not appear on first observation but presented on later observation and extended into the scaffold area. The above process was operated by two investigators to minimize manual counting errors, and the results were incorporated into the data records after both parties agreed.

Blood perfusion of OECs-DBM scaffolds was detected. Under fluorescence microscopy (excitation wavelength 595 nm, emission wavelength 620 nm), four randomly selected fields of view were photographed at the center and border of the scaffolds, respectively. The red fluorescence cumulative fluorescence density values were calculated using Image J software (version 1.0, National Institutes of Health) to reflect the blood perfusion of the OECs-DBM scaffolds.

### HE staining and fluorescence observation of OECs-DBM scaffolds

On day 12 of OECs-DBM scaffold implantation, the scaffolds and its surrounding full-thickness skin within a range of about 0.5 cm were excised, and hard tissue sections were stained with HE staining and fluorescence observation to investigate the fusion mechanism. Briefly, the vascularized observation window was removed from the dorsal surface. The scaffold was removed to an extent of approximately 0.5 cm around the OECs-DBM scaffold, and then the scaffold was cut along with the full skin layer at its base. Paraformaldehyde (4%) was fixed for 2–3 days, and specimens requiring fluorescence observation were fixed in 70% alcohol. Subsequently, the samples were permeabilized, embedded, blocked, sectioned, and mounted. Finally, HE staining of the sections was performed by referring to the kit instructions (Beyotime, China). The distribution of cells in the scaffolds was observed under an optical microscope (Olympus, Tokyo, Japan). Fluorescence microscopy (excitation wavelength 488 nm, emission wavelength 597 nm) to observe the distribution of fluorescent cells in the sections.

### Statistical analysis

SPSS 20.0 statistical software was used for the analysis, and the data were expressed as mean ± standard deviation. After the data were tested for normal distribution and chi-square, *t*-test was used for comparison between two groups, and measurements of the same subject at different observation time points were analyzed using ANOVA with repeated measurement data. Differences were considered statistically significant at *P* < 0.05.

## Result

### Microvessel observation and imaging of dorsal skinfold window-chamber model combined with Texas red-dextran

Our previous studies have shown that *in vitro* dynamic perfusion systems promote the ability of OECs to spread adhesion, proliferation, and three-dimensional tube formation on the DBM. Nevertheless, under this condition, the vascularization process of OECs-DBM scaffolds and the mechanism of their fusion with the vessels in the recipient area are not clear *in vivo*. Accordingly, we intend to observe the role of *in vitro* dynamically perfused OECs-DBM scaffolds by constructing a dorsal skinfold window-chamber model.

All nude mice survived the modeling process and passed the shock period smoothly. The pressure of the vascular observation window was moderate, and the surrounding skin was free of infection and compression necrosis.

**Figure 4.**
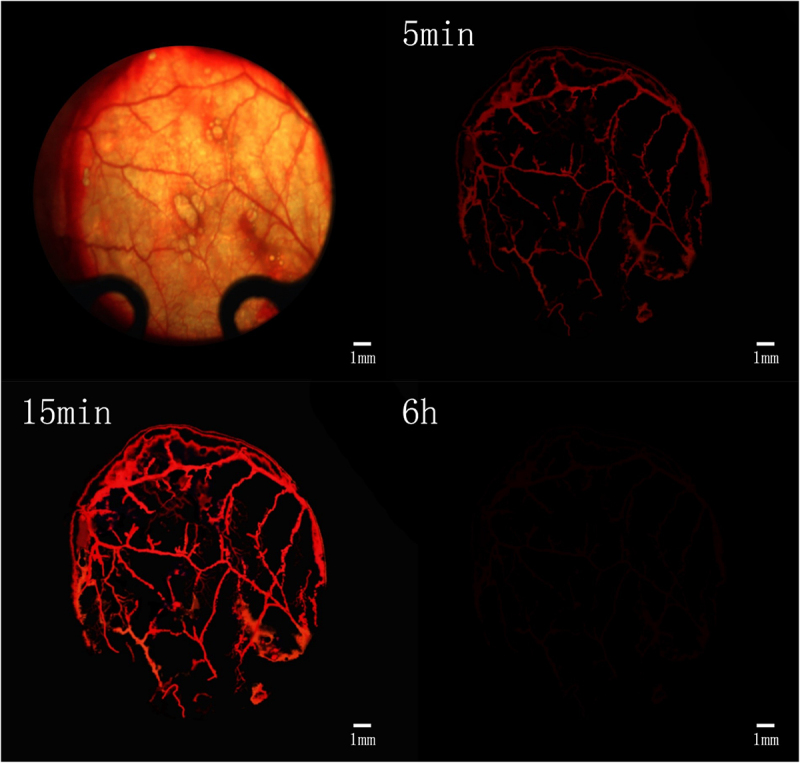
Determination of time window for observation of vascularization. Scale bar: 1 mm.

After tail vein injection of Texas red dextran, the fluorescence microscopy showed that the microvessels were visible after about 5 min, and the vascular paths were basically the same as those under the light microscope (Figure 4). After the injection, the fluorescence brightness peaked about 15 min and lasted for more than 30 min, and then the fluorescence gradually disappeared (Figure 4). At about 6 h after injection, the fluorescence had largely subsided (Figure 4). This prompted us that the observation time of fluorescent microvessels should be ideally 15 min after injection.

**Figure 5.**
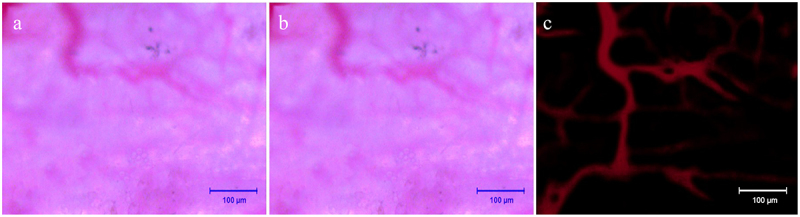
Observation and fluorescent imaging of microvessels. a: Microvessels under light microscopy prior to injection of Texas red-dextran; b: Microvessels under light microscopy after injection of Texas red-dextran; c: Microvessels under fluorescence microscope after injection of Texas red-dextran. Scale bar: 100 μm.

Before and after injection of Texas red-dextran, the image was blurred in the light microscopic field of view, smudges were visible, the image background lacked contrast with the vessels, and the vessels were poorly displayed (Figure 5(a,b)). The fluorescent vessels were clearly visualized with strong contrast to the background, and the vessels were accurately counted, reducing manual counting errors (Figure 5(c)). After statistics, it was found that the diameter of blood vessels that were not imaged under the light microscope was 9.3 ±2.1 μm. This suggests that it is more beneficial to count plasma microvessels and calculate scaffold blood perfusion by this method.

### Dynamic observation of the vascularization process of OECs-DBM scaffolds in vivo

**Figure 6.**
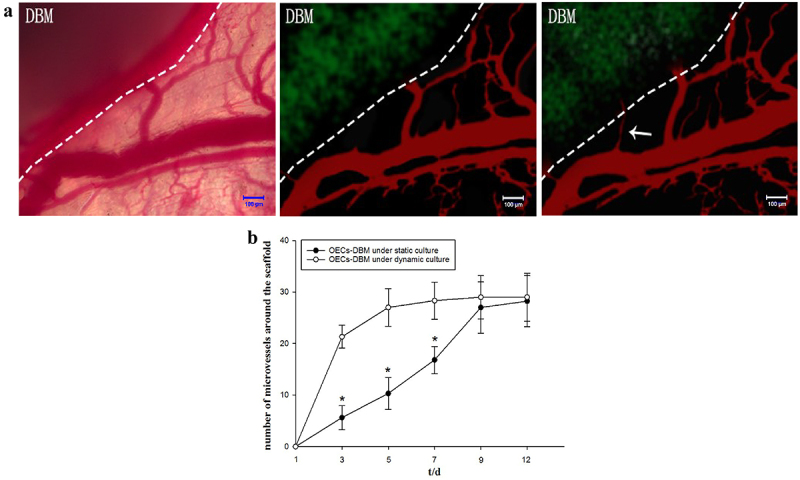
Dynamic observation of vascularization in OECS-DBM complex. a: Ingrowth of recipient microvessels across the verge of OECs-DBM complex. Scale bar: 100 μm. Dot line represents verge of OECs-DBM complex, white arrow represents recipient microvessel grows into OECs-DBM complex. b: Number of ingrown microvessels–time curve. Compare with OECs-DBM scaffold under dynamic culture, *P < 0.05.

Further, we counted new growth into the vessel at the edge of the OECs-DBM scaffold. The result exhibited that the OECs-DBM scaffold gradually grew into microvessels around the recipient area after implantation (Figure 6(a,b)). With the extension of culture time, the number of ingrown microvessels gradually increased (Figure 6(b)). In the first week, the number of newly ingrown microvessels was greater in the dynamic perfusion compared with the static culture group (Figure 6(b), p < 0.05). Since then, the rate of increase in the number of newly grown vessels in the dynamic perfusion group slowed down, while that in the static culture group continued to increase slowly, which eventually reached a level comparable to that of the dynamic perfusion group.

**Figure 7.**
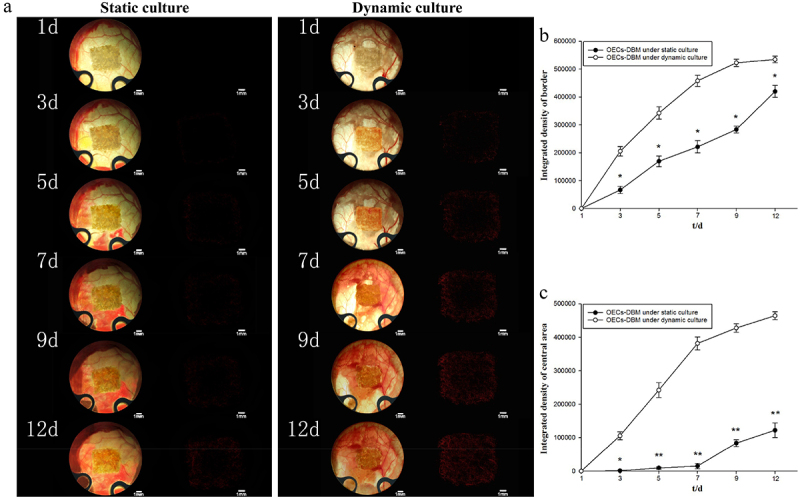
OECS-DBM complex was observed in static culture and dynamic culture group. a: *In vivo* observation and fluorescent imaging of OECs-DBM complex under static culture (left) and dynamic culture (right). Scale bar: 1 mm. b: Integrated density of scaffold border–time curve. c: Integrated density of scaffold central area–time curve. Compare with OECs-DBM scaffold under dynamic culture, *P < 0.05, **P < 0.01.

The OECs-DBM scaffold was seen to be white when it was first implanted and then gradually turned yellow. The skin and subcutaneous tissue around the window regenerated and approached the scaffold within the observation window, and the surrounding vessels regenerated and gradually grew into the scaffold. At the end of the observation, the scaffold turned red, and new vessels grew into the scaffold border and central area surface (Figure 7(a)). Statistical integrated density values in the scaffold edge region exhibited that the scaffold in the dynamic perfusion group was higher than that in the static culture group at all observed time points (Figure 7(b), p < 0.05). The difference in the integrated density values of fluorescence in the central region between the two groups of OECs-DBM scaffolds was more significant (Figure 7(c), p < 0.01). The integrated density values of fluorescence in the central region of the scaffold in the static culture group increased extremely slowly in the first week, and by the end of the observation, and the fluorescence was still extremely weak under the fluorescence microscope. The integrated density values of fluorescence in the central region of the scaffold in the dynamic perfusion group rose to a higher level in the first week, and at the end of the observation, the fluorescence density observed under fluorescence microscopy was almost the same as that at the edge of the scaffold.

### HE staining and fluorescence observation of the OECs-DBM scaffold and its surrounding tissues

**Figure 8.**
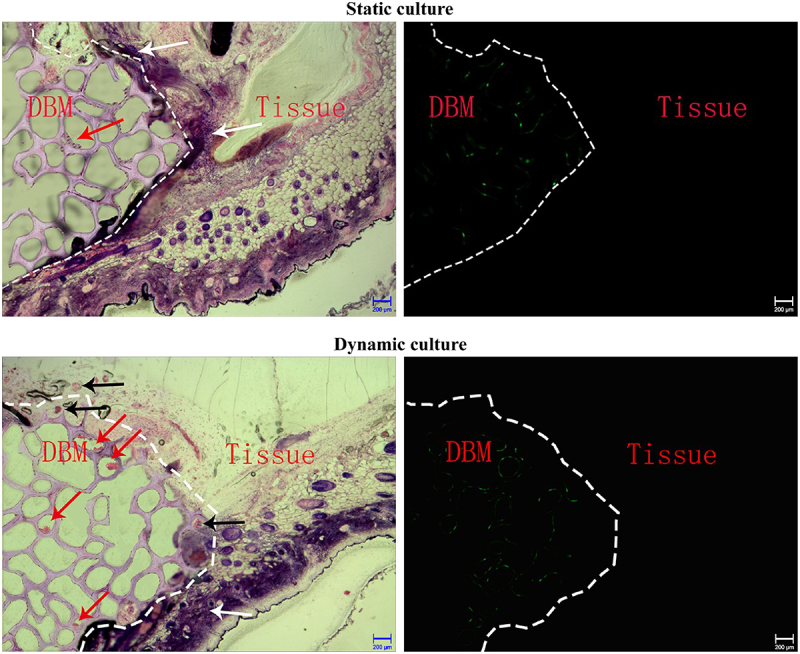
HE staining and fluorescent imaging of OECs-DBM scaffold slice under static culture and dynamic culture. Dot line represents verge of scaffold, red arrow represents OECs lumps, white arrow represents inflammatory cells, black arrow represents microvessels from recipient. Scale bar: 200 μm.

In the static culture group, HE results displayed that the cells on the OECs-DBM scaffold were unevenly distributed and formed cell clumps. There was no or occasional ring-shaped tubular structure of intact cells in the pores of the scaffold, and inflammatory cell infiltration was seen (Figure 8). Under the fluorescence microscope, a small amount of green fluorescence was scattered on the scaffold, and no green fluorescence was seen in the tissue surrounding the scaffold (Figure 8). In the dynamic perfusion group, the cells on the OECs-DBM scaffold were evenly distributed, and many intact cells were seen in the pores of the scaffold, with annular tubular structures and a small amount of inflammatory cell infiltration (Figure 8). Under the fluorescence microscope, more green fluorescence was seen on the scaffold, and green fluorescent cells were seen to form a complete annular tubular structure, and there is no green fluorescence in the tissues surrounding the scaffold (Figure 8).

## Discussion

One of the current areas of research focus in bone tissue engineering is vascularization techniques. The key to survival and function of tissue-engineered bone after implantation in the body is whether it can quickly obtain sufficient blood perfusion. To carry out research on provascularization of tissue-engineered bone, it is necessary to establish objective methods for the detection and observation of vascularization. Currently, the two main types of observation methods for microvessels are *in vivo* specimens and *in vivo* observations. The *in* vivo method is only applicable to the observation of microvasculature in dead or isolated specimens, neither of which reflects the microvascular condition in the normal physiological state. Notably, Sandison et al. first applied the animal dorsal skinfold window-chamber model to observe living tissues [24]. Tissue engineering has developed rapidly in the past 20 years, and many experimental studies have used the dorsal skinfold window-chamber model to dynamically observe the histocompatibility, inflammatory response, and vascularization process of receptors to different biomaterials in real time [25–27]. Because angiogenesis is a dynamic process, a major advantage of the dorsal skinfold window-chamber model is the ability to monitor the angiogenesis process *in vivo* continuously and dynamically for up to several weeks. In addition, at the end of the experiment, tissue samples can be easily excised for many tests such as histology, immunohistochemistry, and molecular biology.

Based on this, we constructed a dorsal skinfold window-chamber model in nude mice. Moreover, for the requirement of accuracy in vessel counts and the need to calculate blood perfusion in OECs-DBM scaffolds, we gave tail vein injections of Texas Red-Dextran for labeling plasma in nude mice. Our results indicate that the method is more accurate and clear for counting plasma microvessels and calculating scaffold blood perfusion. Since dextran prevents erythrocyte and platelet aggregation, reduces blood viscosity, and improves microcirculation, it may have an effect on microvascular counts. We observed and compared microvascular images in the same field of view before and after the injection, and found that this dose of dextran did not affect microvascular counts. After injections of Texas Red-Dextran, microvessels not observed under the light microscope could be seen under the fluorescence microscope, presumably because the microvessels were too fine in diameter and could not be easily distinguished from the background. It is obvious that the model injected with Texas Red-Dextran observes the fluorescent blood vessels clearly, with a strong contrast with the background, and the blood vessels are counted accurately, which reduces the error of the researchers’ manual counting, and has more advantages than simply observing under a light microscope.

In this study, the tissue-engineered product used was an *in vitro* dynamically perfusion-treated prevascularized OECs-DBM scaffold, and its pro-vascularization ability after implantation was compared with that of a statically perfused OECs-DBM scaffold. We have previously shown that a dynamic perfusion system that imparts continuous fluid shear stress to OECs on DBM scaffolds can promote spreading adhesion, proliferation, differentiation to mature endothelial cells, and three-dimensional tube formation in stromal pores via the PI3K/Akt/mTOR signaling pathway, promoting in vitro prevascularization of DBM scaffolds [22]. The result revealed that, after implantation of OECs-DBM scaffolds with static and dynamic perfusion cultures, the number of ingrown vessels and the blood perfusion obtained were higher than those of the static perfusion group, especially the blood perfusion obtained in the central area of the scaffold increased in the first week. In addition, HE staining results indicated the presence of recipient erythrocytes in the scaffold pores. All of these suggested that the vessels on *in vivo* dynamically perfusion-treated prevascularized OECs-DBM scaffold were able to inosculation with the recipient vessels in a certain way to rapidly establish a blood supply connection, allowing tissue-engineered bone to obtain a faster and better blood supply to the recipient region *in vivo* and improving the survival rate of the cells on the scaffold.

There are few studies on the mechanism of vascular inosculation. In contrast, the study of the mechanism of inosculation of tissue-engineered products with recipient vessels has not been reported, which is one of the innovations of this study. It is generally accepted that there are two types of inosculations between the recipient vessel and the graft vessel: internal inosculation and external inosculation. Some studies suggest that vascular inosculation occurs within the graft and that the mechanism is intra-graft vascular regression, with the recipient vascular network growing in along the graft and fusing with it [28,29]. In contrast, recent findings support an external inosculation, in which the vessel of the graft buds and grows toward the recipient vessel, with the vascular inosculation process occurring outside the graft [30–33]. Some studies have even suggested that the two vascular inosculation modes occur simultaneously and coexist [34]. The experimental studies supporting external inosculation are basically *in vivo* prevascularization methods to construct tissue-engineered scaffolds or simply tissue or cell masses of newborn animals enriched with various pro-vascular growth-related factors and active substances. For example, nano-hydroxyapatite/polyether polyurethane scaffolds were prevascularized *in vivo* and cultured with VEGF-rich Matrigel gel [30]. *In vivo* prevascularized PLGA scaffold [35]. VEGF- and BFGF-rich adipose tissue-derived vascular fragment composite nano-hydroxyapatite/polyether polyurethane scaffold [36]. Cellular spheroids are constructed from human osteoblasts, dermal microvascular endothelial cell and dermal fibroblasts [33]. The grafts used in the experimental studies supporting internal inosculation were normal skin tissue [28,29]. The only study that suggested the coexistence of both fusion modalities was the implantation of *in vivo* prevascularized polyether polyurethane scaffolds into nude mice after 3 days of in vitro incubation in DMEM medium, which was found to increase the expression of VEGF on the scaffolds, accelerating the process of vascular fusion and the coexistence of both fusion modalities [34]. Combined with the results of this study, we suggest that the *in vitro* dynamically perfused OECs-DBM scaffold preconfigured vessels are fused to the recipient vessels in an internal inosculation to obtain blood supply. Because, no green fluorescent cells of OECs were seen outside the scaffold, and the complete luminal structure of the vessel composed of OECs was visible inside the scaffold, and receptor erythrocytes were visible in it.

The results of this experimental study were positive. Nevertheless, we believe that further optimization is needed. On the one hand, the rate of vascular inosculation also depends on the angiogenic process, such as the angiogenic activity of the preconstituted and recipient vessels. Therefore, vascular inosculation can be optimized by further improving the angiogenic activity of the preconstituted vessels or the recipient tissue, such as VEGF gene transfection of OECs. On the other hand, it has been demonstrated that the inosculation of preconfigured vessels with vascular buds at both ends of the recipient tissue is mainly dependent on the balance between Notch and Wnt signaling pathways, which control the stability of the neovascular network [37]. Therefore, it can also be further optimized by the modulation of signaling pathways. Notably, vascular inosculation can also be combined with seed cell pretreatment methods in the future to enhance the ischemic-hypoxic tolerance of seed cells within the stent during the initial implantation phase [38].

## Conclusion

In summary, this study successfully constructed a dorsal skinfold window-chamber model and established its method for counting microvessels and calculating scaffold blood perfusion volume in combination with Texas-red dextran-labeled plasma. In addition, we demonstrated that *in vitro* dynamic perfusion culture of prevascularized OECs-DBM scaffolds resulted in faster and more adequate fusion with the recipient vessels in an internal inosculation to obtain blood perfusion to the recipient area.
